# Trauma care: Finding a better way

**DOI:** 10.1371/journal.pmed.1002350

**Published:** 2017-07-18

**Authors:** Hasan B. Alam

**Affiliations:** Department of Surgery, University of Michigan, Ann Arbor, Michigan, United States of America

## Abstract

In a Perspective, Hasan Alam discusses emerging treatment approaches in trauma care.

“There’s a way to do it better—find it.”—Thomas Edison (1847–1931)

## Changing patterns of deaths due to injuries

Since the second half of the 20th century, we have seen revolutionary changes in medicine, and trauma care is no exception. Injuries remain the primary cause of death for Americans under 46 years of age [1], but the patterns are changing. Today, in massively bleeding patients without head injuries, mortality beyond the first 24 hours is under 10% [2]. Unfortunately, the area in which we have failed to make a difference is the period immediately following the injury, including the prehospital phase. The majority of deaths in this period are due to hemorrhage and/or traumatic brain injury (TBI). Bleeding is the more treatable of these 2 causes of death, which makes it the number 1 cause of preventable deaths. Despite numerous advances in trauma care, a recent multinational trial of more than 20,000 patients [3] demonstrated that most deaths occur within a few hours of injury, with <2.5% of the injured succumbing to multiple organ failure. Similarly, in combat, 87% of battlefield deaths occur before reaching a medical facility [4]; nearly a quarter of these injuries are considered potentially survivable, and this category is largely (91%) made up of deaths due to bleeding. Thus, the current goal of early care is to keep patients alive long enough to be evacuated to higher echelons of care for definitive treatment.

## Future directions

In the not-too-distant future, trauma care is likely to be very different from the current practice. In addition to early hemorrhage control and damage control resuscitation, we are also likely to see the following:

- The emergence of specific prosurvival drugs that can be given in the prehospital setting to keep injured people alive long enough to permit transfer to higher levels of care.
- Early (prehospital) use of preserved plasma products, platelets, and red blood cells.
- Availability of blood “farming” to eliminate the logistical barriers to supply, in which immortalized cell lines could efficiently generate red blood cells, in vitro, in a sustainable fashion [5].
- Development of safe and effective nonblood oxygen-carrying fluids that can be easily administered.
- Temporary use of hypothermia or hibernation strategies for patients with potentially survivable injuries but who need more time for surgery or transfer.
- Individualized therapy, also known as precision medicine, with administration of agents based upon the individual’s specific needs.
- Monitoring of response to therapy that goes beyond the measurement of basic physiology by looking at key molecular and cellular disturbances.

Many of these novel treatments are already at the cusp of clinical reality, and I discuss 2 examples here.

## Pharmacological treatment to create a prosurvival phenotype

We know that shock can disrupt cellular acetylation homeostasis by altering the balance between the histone deacetylase (HDAC) and histone acetyltransferase (HAT) families of enzymes [6]. Valproic acid (VPA), a commonly used anti-seizure medicine, is a nonselective histone deacetylase inhibitor (HDACI) when given in larger doses (higher than the commonly used anti-seizure dose) and can cause rapid and reversible acetylation of numerous nuclear and cytoplasmic proteins to create an anti-inflammatory and prosurvival phenotype [6,7]. In fact, a single dose of VPA, even in the absence of conventional resuscitation strategies, has been shown to improve survival and mitigate organ damage in models of lethal hemorrhage [8], poly-trauma [9,10], septic shock [11], ischemia-reperfusion injury [12], and TBI [13]. Using a variety of in vitro and in vivo models, we have also identified multiple molecular pathways that are modulated by VPA treatment [6,7]. These findings are potentially clinically relevant, as we have shown that expression profiles of various HDACs in circulating cells are associated with differences in clinical outcomes in trauma patients [14]. Additionally, tissues obtained from trauma patients display decreased acetylation, which can be rapidly normalized (ex vivo) with HDACI treatment [15].

These promising preclinical results have allowed us to perform a Phase I clinical trial of VPA for the treatment of hemorrhage (ClinicalTrials.gov, NCT01951560), and Phase II and III clinical trials are expected to follow. This pharmacological approach is equally effective when hemorrhage is complicated by severe TBI. Working with clinically relevant large-animal models of TBI and hemorrhagic shock (HS), we have shown that a single dose of VPA can attenuate brain lesion size, inflammation, and edema within 6 hours of treatment [16]. Treatment with fresh frozen plasma (FFP) has also been shown to be very effective, both as a monotherapy [17] and in combination with VPA—resulting in a synergistic effect [18]. VPA treatment up-regulates expression of beneficial genes in the injured brain [19,20], modulates posttraumatic brain metabolism [21], and improves long-term neurological recovery and healing [22] in clinically relevant models of TBI combined with HS.

In large animals, a single dose of VPA (150 mg/kg) restores acetylation and attenuates cell death, as evidenced by smaller brain lesion size and edema [22]. In the Phase I VPA trial, we found that doses of 130 mg/kg and 140 mg/kg were well tolerated in humans with no dose-limiting toxicities [23], and high-throughput proteomic analysis (in the 120 mg/kg cohort) has revealed 140 unique differentially expressed protein domains [24]. VPA also reversibly alters nucleosome topography [25] and activates neurogenic transcriptional programs in the adult human brain following traumatic injury [26]. The fact that VPA has been in clinical use for >40 years, is relatively inexpensive (approximately $40 per dose), has no special storage needs, and is easy to administer justifies its development as a “bridge therapy” for austere field care environments.

## Therapeutic hypothermia

Often, the underlying injuries are reparable, but a patient dies of irreversible shock or severe brain damage. In this setting, strategies to maintain cerebral and cardiac viability long enough to gain control of hemorrhage and restore intravascular volume could be lifesaving. This requires an entirely new approach to the problem, with emphasis on rapid total body preservation, repair of injuries during metabolic arrest, and controlled resuscitation, the process of which has been termed emergency preservation and resuscitation (EPR). Currently, hypothermia is the most effective method for preserving cellular viability during prolonged periods of ischemia [27]. It is clear from canine models that rapid induction of deep/profound hypothermia (<15°C) can improve an otherwise dismal outcome after exsanguinating cardiac arrest [28,29]. Our team has used clinically realistic large-animal models of lethal vascular injuries and soft tissue trauma to demonstrate that profound hypothermia can be induced through an emergency thoracotomy approach for total body protection, with excellent long-term survival and no neurological damage or significant organ dysfunction, and that otherwise lethal vascular injuries, above and below the diaphragm, can be repaired under hypothermic arrest with greater than 75% long-term survival [30].

Subsequent studies have determined that, to achieve the best results, profound hypothermia must be induced rapidly (2°C/min) and reversed at a slower rate (0.5°C/min) [31,32]. The optimal depth of hypothermia is 10°C, and decreasing the temperature to ultraprofound levels (5°C) may worsen the outcome [33]. If hypothermia is induced appropriately, the safe duration of total body preservation is around 60 minutes [34], and there is no increase in postoperative bleeding or septic complications in the setting of solid organs and bowel injuries [35]. This approach may have significant implications not only for treating traumatic injuries but also for preserving organs for transplant [36]. The expertise to preserve the viability of key organs during repair of otherwise lethal injuries is now clearly available [27]. Although there are logistical challenges to the adoption of EPR in trauma practice [37], a prospective multi-institutional trial is already underway to establish its feasibility [38].

## Discussion

To save the numerous lives that are lost to hemorrhage and TBI every day, new therapeutic approaches are needed. There is clearly room for improvement. According to the United States Centers for Disease Control and Prevention, National Center for Injury Prevention and Control, about a quarter (27 million in 2013) of all emergency department visits are due to injuries [39], resulting in 3 million hospitalizations and nearly 193,000 deaths—1 person every 3 minutes [40]. As opposed to cancer, cardiovascular disease, and stroke, injuries disproportionally strike people in the prime of their lives. In fact, 59% of all deaths among people 1–44 years of age in the US are due to injuries, which is a higher proportion than all noncommunicable and infectious diseases combined. In 2013, the total cost of injuries in the US was estimated to be $671 billion [41,42]. Globally, according to the World Health Organization, injuries kill more than 5 million people each year, which is nearly 1.7 times the number of fatalities from malaria, tuberculosis, and HIV combined [43]. Many resource-constrained countries lack established trauma systems resulting in prolonged prehospital times, and the healthcare facilities lack resources that are taken for granted in resource-rich countries (e.g., well-stocked blood banks, intensive care units, advanced radiology, sophisticated monitoring tools). Arguably, easy-to-administer, cost-effective pharmacological interventions are logistically a much more attractive option in these resource-constrained settings, as we have already seen with tranexamic acid in the CRASH-2 trial [3]. Similarly, the battlefield environment is another place where rugged, easy-to-use interventions that can keep an injured person alive long enough to get evacuated to specialized care can save numerous lives. Many such technologies are potentially within our grasp; we just need to be open to change.

## Abbreviations

EPR: emergency preservation and resuscitation
FFP: fresh frozen plasma
HAT: histone acetyltransferase
HDAC: histone deacetylase
HDACI: histone deacetylase inhibitor
HS: hemorrhagic shock
TBI: traumatic brain injury
VPA: valproic acid

## References

[1] Rhee P, Joseph B, Pandit V, Aziz H, Vercruysse G, Kulvatunyou N, et al Increasing trauma deaths in the United States. Ann Surg. 2014 7;260(1):13–21. doi: 10.1097/SLA.0000000000000600 24651132 10.1097/SLA.0000000000000600

[2] Shackford SR, Mackersie RC, Holbrook TL, Davis JW, Hollingsworth-Fridlund P, Hoyt DB, et al The epidemiology of trauma death: a population based analysis. Arch Surg. 1993;128:571–5. 8489391 10.1001/archsurg.1993.01420170107016

[3] CRASH-2 trial collaborators. Effects of tranexamic acid on death, vascular occlusive events, and blood transfusion in trauma patients with significant haemorrhage (CRASH-2): a randomised, placebo-controlled trial. Lancet. 2010;376:23–32. doi: 10.1016/S0140-6736(10)60835-5 20554319 10.1016/S0140-6736(10)60835-5

[4] Eastridge BJ, Mabry RL, Seguin P, Cantrell J, Tops T, Uribe P, et al Death on the battlefield (2001–2011): implications for the future of combat casualty care. J Trauma Acute Care Surg. 2012;73(6 suppl 5):S431–S437. doi: 10.1097/TA.0b013e3182755dcc 23192066 10.1097/TA.0b013e3182755dcc

[5] Trakarnsanga K, Griffiths RE, Wilson MC, Blair A, Satchwell TJ, Meinders M, et al An immortalized adult human erythroid line facilitates sustainable and scalable generation of functional red cells. Nat Commun. 2017 3 14;8:14750 doi: 10.1038/ncomms14750 28290447 10.1038/ncomms14750 PMC5355882

[6] Li Y, Alam HB. Creating a pro-survival and anti-inflammatory phenotype by modulation of acetylation in models of hemorrhagic and septic shock. Adv Exp Med Biol. 2012;710:107–33. doi: 10.1007/978-1-4419-5638-5_11 22127890 10.1007/978-1-4419-5638-5_11 PMC4894314

[7] Halaweish I, Nikolian V, Georgoff P, Li Y, Alam HB. Creating a "Prosurvival Phenotype" Through Histone Deacetylase Inhibition: Past, Present, and Future. Shock. 2015;44 Suppl 1:6–16. 25565645 10.1097/SHK.0000000000000319 PMC4492909

[8] Shults C, Sailhamer EA, Li Y, Liu B, Tabbara M, Butt MU, et al Surviving blood loss without fluid resuscitation. J Trauma. 2008;64:629–38. doi: 10.1097/TA.0b013e3181650ff3 18332802 10.1097/TA.0b013e3181650ff3

[9] Alam HB, Shuja F, Butt MU, Duggan M, Li Y, Zacharias N, et al Surviving blood loss without blood transfusion in a swine poly-trauma model. Surgery. 2009;146:325–33. doi: 10.1016/j.surg.2009.04.007 19628092 10.1016/j.surg.2009.04.007

[10] Alam HB, Hamwi KB, Duggan M, Fikry K, Lu J, Fukudome EY, et al Hemostatic and pharmacologic resuscitation: results of a long-term survival study in a swine polytrauma model. J Trauma. 2011;70:636–45. doi: 10.1097/TA.0b013e31820d0dcc 21610354 10.1097/TA.0b013e31820d0dcc

[11] Liu Z, Li Y, Chong W, Deperalta DK, Duan X, Liu B, et al Creating a prosurvival phenotype through a histone deacetylase inhibitor in a lethal two-hit model. Shock. 2014;41:104–8. doi: 10.1097/SHK.0000000000000074 24430491 10.1097/SHK.0000000000000074 PMC4822203

[12] Kim K, Li Y, Jin G, Chong W, Liu B, Lu J, et al Effect of valproic acid on acute lung injury in a rodent model of intestinal ischemia reperfusion. Resuscitation. 2012;83:243–8. doi: 10.1016/j.resuscitation.2011.07.029 21824465 10.1016/j.resuscitation.2011.07.029 PMC3242830

[13] Jepsen CH, deMoya MA, Perner A, Sillesen M, Ostrowski SR, Alam HB, et al Effect of valproic acid and injury on lesion size and endothelial glycocalyx shedding in a rodent model of isolated traumatic brain injury. J Trauma Acute Care Surg. 2014;77:292–7. doi: 10.1097/TA.0000000000000333 25058256 10.1097/TA.0000000000000333

[14] Sillesen M, Bambakidis T, Dekker SE, Fabricius R, Svenningsen P, Bruhn PJ, et al Histone deactylase gene expression profiles are associated with outcomes in blunt trauma patients. J Trauma Acute Care Surg. 2016;80:26–33. doi: 10.1097/TA.0000000000000896 26517778 10.1097/TA.0000000000000896

[15] Sailhamer EA, Li Y, Smith EJ, Shuja F, Shults C, Liu B, et al Acetylation: a novel method for modulation of the immune response following trauma/hemorrhage and inflammatory second hit in animals and humans. Surgery. 2008;144:204–16. doi: 10.1016/j.surg.2008.03.034 18656627 10.1016/j.surg.2008.03.034

[16] Jin G, Duggan M, Imam A, Demoya MA, Sillesen M, Hwabejire J, et al Pharmacologic resuscitation for hemorrhagic shock combined with traumatic brain injury. J Trauma Acute Care Surg. 2012;73:1461–70. doi: 10.1097/TA.0b013e3182782641 23188239 10.1097/TA.0b013e3182782641

[17] Jin G, DeMoya MA, Duggan M, Knightly T, Mejaddam AY, Hwabejire J, et al Traumatic brain injury and hemorrhagic shock: evaluation of different resuscitation strategies in a large animal model of combined insults. Shock. 2012;38:49–56. doi: 10.1097/SHK.0b013e3182574778 22575994 10.1097/SHK.0b013e3182574778

[18] Imam AM, Jin G, Duggan M, Sillesen M, Hwabejire JO, Jepsen CH, et al Synergistic effects of fresh frozen plasma and valproic acid treatment in a combined model of traumatic brain injury and hemorrhagic shock. Surgery. 2013;154:388–96. doi: 10.1016/j.surg.2013.05.008 23889966 10.1016/j.surg.2013.05.008

[19] Bambakidis T, Dekker SE, Sillensen M, Liu B, Johnson CN, Jin G, et al Resuscitation With Valproic Acid Alters Inflammatory Genes in a Porcine Model of Combined Traumatic Brain Injury and Hemorrhagic Shock. J Neurotrauma. 2016 4 8;33:1514–21. doi: 10.1089/neu.2015.4163 26905959 10.1089/neu.2015.4163

[20] Dekker SE, Bambakidis T, Sillesen M, Liu B, Johnson CN, Jin G, et al Effect of pharmacologic resuscitation on the brain gene expression profiles in a swine model of traumatic brain injury and hemorrhage. J Trauma Acute Care Surg. 2014;77:906–12; doi: 10.1097/TA.0000000000000345 25051383 10.1097/TA.0000000000000345

[21] Hwabejire JO, Jin G, Imam AM, Duggan M, Sillesen M, Deperalta D, et al Pharmacologic modulation of cerebral metabolic derangement and excitotoxicity in a porcine model of traumatic brain injury and hemorrhagic shock. Surgery. 2013;154:234–43. doi: 10.1016/j.surg.2013.04.008 23889951 10.1016/j.surg.2013.04.008

[22] Halaweish I, Bambakidis T, Chang Z, Wei H, Liu B, Li Y, et al Addition of low-dose valproic acid to saline resuscitation provides neuroprotection and improves long-term outcomes in a large animal model of combined traumatic brain injury and hemorrhagic shock. J Trauma Acute Care Surg. 2015;79:911–9. doi: 10.1097/TA.0000000000000789 26680134 10.1097/TA.0000000000000789

[23] Georgoff PE, Nikolian VC, Bonham T, Pai MP, Tafatia C, Halaweish I, et al Safety and Tolerability of Intravenous Valproic Acid in Healthy Subjects: A Phase I Dose-Escalation Trial. Clin Pharmacokinet. 2017 5 11 doi: 10.1007/s40262-017-0553-1 28497259 10.1007/s40262-017-0553-1 PMC5699961

[24] Georgoff P, Halaweish I, Nikolian V, Higgins G, Bonham T, Tafatia C, et al Alterations in the human proteome following administration of valproic acid. J Trauma Acute Care Surg. 2016;81(6):1020–1027. doi: 10.1097/TA.0000000000001249 27602906 10.1097/TA.0000000000001249

[25] Higgins GA, Allyn-Feuer A, Georgoff P, Nikolian V, Alam HB, Athey BD. Mining the topography and dynamics of the 4D Nucleome to identify novel CNS drug pathways. Methods. 2017 4 4. pii:S1046-2023(16)30444-3. 10.1016/j.ymeth.2017.03.012 28385536

[26] Higgins GA, Georgoff P, Nikolian V, Allyn-Feuer A, Pauls B, Higgins R, et al Network Reconstruction Reveals that Valproic Acid Activates Neurogenic Transcriptional Programs in Adult Brain Following Traumatic Injury. Pharm Res. 2017 3 7 doi: 10.1007/s11095-017-2130-6 28271248 10.1007/s11095-017-2130-6 PMC5498621

[27] Alam HB, Pusateri AE, Kindzelski A, Egan D, Hoots K, Andrews MT, et al; HYPOSTAT workshop participants. Hypothermia and hemostasis in severe trauma: A new crossroads workshop report. J Trauma Acute Care Surg. 2012 10;73(4):809–17. doi: 10.1097/TA.0b013e318265d1b8 23026915 10.1097/TA.0b013e318265d1b8

[28] Behringer W, Safar P, Wu X, Kentner R, Radovsky A, Kochanek PM, et al Survival without brain damage after clinical death of 60–120 minutes in dogs using suspended animation by profound hypothermia. Crit Care Med. 2003;31:1523–31. doi: 10.1097/01.CCM.0000063450.73967.40 12771628 10.1097/01.CCM.0000063450.73967.40

[29] Taylor MJ, Bailes JE, Elrifai AM, Shih SR, Teeple E, Leavitt ML, et al A new solution for life without blood: asanguineous low-flow perfusion of a whole body perfusate during 3 hours of cardiac arrest and profound hypothermia. Circulation. 1995;91:431–44. 7805248 10.1161/01.cir.91.2.431

[30] Alam HB, Bowyer MW, Koustova E, Gushchin V, Anderson D, Stanton K, et al Learning and memory is preserved following induced asanguineous hyperkalemic hypothermic arrest in a swine model of traumatic exsanguination. Surgery. 2002;132:278–88. 12219024 10.1067/msy.2002.125787

[31] Alam HB, Chen Z, Honma K, Koustova E, Querol RI, Jaskille A, et al The rate of induction of hypothermic arrest determines the outcome in a swine model of lethal hemorrhage. J Trauma. 2004;57:961–9. 15580018 10.1097/01.ta.0000149549.72389.3f

[32] Alam HB, Rhee P, Honma K, Chen H, Ayuste EC, Lin T, et al Does the rate of rewarming from profound hypothermic arrest influences the outcome in a swine model of lethal hemorrhage. J Trauma. 2006;60:134–46. doi: 10.1097/01.ta.0000198469.95292.ec 16456447 10.1097/01.ta.0000198469.95292.ec

[33] Alam HB, Chen Z, Li Y, Velmahos G, DeMoya M, Keller CE, et al Profound hypothermia is superior to ultra-profound hypothermia in improving survival in a swine model of lethal injuries. Surgery. 2006;140:307–14. doi: 10.1016/j.surg.2006.03.015 16904984 10.1016/j.surg.2006.03.015

[34] Alam HB, Duggan M, Li Y, Spaniolas K, Liu B, Tabbara M, et al Putting life on hold-for how long? Profound hypothermic cardiopulmonary bypass in a swine model of complex vascular injuries. J Trauma. 2008;64:912–22. doi: 10.1097/TA.0b013e3181659e7f 18404056 10.1097/TA.0b013e3181659e7f

[35] Sailhamer EA, Chen Z, Ahuja N, Velmahos GC, de Moya M, Rhee P, et al Profound hypothermic cardiopulmonary bypass facilitates survival without a high complication rate in a swine model of complex vascular, splenic and colon injuries. J Am Coll Surg. 2007;204:642–53. doi: 10.1016/j.jamcollsurg.2007.01.017 17382224 10.1016/j.jamcollsurg.2007.01.017

[36] Taylor MJ, Rhee P, Chen Z, Alam HB. Design of preservation solutions for universal tissue preservation in vivo: demonstration of efficacy in pre-clinical models of profound hypothermic cardiac arrest. Transplant Proc. 2005;37:303–7. doi: 10.1016/j.transproceed.2004.12.024 15808626 10.1016/j.transproceed.2004.12.024

[37] Alam HB. Translational barriers and opportunities for emergency preservation and resuscitation in severe injuries. Br J Surg. 2012 1;99 Suppl 1:29–39. 10.1002/bjs.7756 22441853

[38] Tisherman SA, Alam HB, Rhee PM, Scalea TM, Drabek T, Forsythe RM, et al Development of the Emergency Preservation and Resuscitation for Cardiac Arrest from Trauma (EPR-CAT) Clinical Trial. J Trauma Acute Care Surg. 2017 5 22 doi: 10.1097/TA.0000000000001585 28538639 10.1097/TA.0000000000001585

[39] Centers for Disease Control and Prevention, National Center for Injury Prevention and Control. Web-based Injury Statistics Query and Reporting System (WISQARS) Nonfatal Injury Data. (2015). http://www.cdc.gov/injury/wisqars/nonfatal.html. Accessed 25 April 2017.

[40] Centers for Disease Control and Prevention, National Center for Injury Prevention and Control. Web-based Injury Statistics Query and Reporting System (WISQARS) Fatal Injury Data. (2015). http://www.cdc.gov/injury/wisqars/fatal.html. Accessed 25 April 2017.

[41] Florence C, Simon T, Haegerich T, Luo F, Zhou C. Estimated Lifetime Medical and Work Loss Costs of Fatal Injuries—United States 2013. MMWR Morb Mortal Wkly Rep. 2015;64(38):1074–7. http://www.cdc.gov/mmwr/preview/mmwrhtml/mm6438a4.htm?s_cid=mm6438a4_w. Accessed 25 April 2017. doi: 10.15585/mmwr.mm6438a4 26421530 10.15585/mmwr.mm6438a4

[42] Florence C, Haegerich T, Simon T, Zhou C, Luo F. Estimated Lifetime Medical and Work Loss Costs of Emergency Department Treated Nonfatal Injuries—United States 2013. MMWR Morb Mortal Wkly Rep. 2015;64(38):1078–82. http://www.cdc.gov/mmwr/preview/mmwrhtml/mm6438a5.htm?s_cid=mm6438a5_w. Accessed 25 April 2017. doi: 10.15585/mmwr.mm6438a5 26421663 10.15585/mmwr.mm6438a5

[43] World Health Organization. Injuries and violence: the facts 2014. http://www.who.int/violence_injury_prevention/media/news/2015/Injury_violence_facts_2014/en/. Accessed 25 April 2017.

